# Optimization of Thermal Control Design for Aerial Reflective Opto-Mechanical Structure

**DOI:** 10.3390/s24041194

**Published:** 2024-02-12

**Authors:** Huilin Wang, Yun Zhou, Xiaocun Jiang, Xiaozhou Zuo, Ming Chen

**Affiliations:** Xi’an Institute of Applied Optics, Xi’an 710118, China

**Keywords:** opto-mechanical structure, optimized thermal control, temperature uniformity, image quality

## Abstract

To improve the adaptability of aerial reflective opto-mechanical structures (mainly including the primary mirror and secondary mirror) to low-temperature environments, typically below −40 °C, an optimized thermal control design, which includes passive insulation and temperature-negative feedback-variable power zone active heating, is proposed. Firstly, the relationship between conventional heating methods and the axial/radial temperature differences of mirrors with different shapes is analyzed. Based on the heat transfer analyses, it is pointed out that optimized thermal control design is necessary to ensure the temperature uniformity of the fused silica mirror, taking into account the temperature level when the aerial electro-optics system is working in low-temperature environments. By adjusting the input voltage based on the measured temperature, the heating power of the subregion is changed accordingly, so as to locally increase or decrease the temperature of the mirrors. The thermal control scheme ensures that the average temperature of the mirror fluctuates slowly and slightly around 20 °C. At the same time, the temperature differences within a mirror and between the primary mirror and the secondary mirror can be controlled within 5 °C. Thereby, the resolution of EO decreases by no more than 11.4%.

## 1. Introduction

To obtain clear images, spacecraft and aircraft are equipped with electro-optics (EO) systems [1,2]. Generally, EO systems mainly include electronic units, opto-mechanical structures, and imaging sensors, which are mounted inside the cabin. For large EO, the opto-mechanical structure is often designed as a reflective type, and the main optical components include a primary mirror and secondary mirror [3,4].

To improve the ability to clearly image distant targets, it is necessary to have a larger aperture for the primary mirror and secondary mirror, while ensuring a minimal wavefront error (WFE) on the reflective surface [5]. The WFE is impacted by the manufacturing and assembly technology and also varies with environmental factors [6,7]. Among various environmental factors, the influence of the temperature is enormous. When the temperature changes, the curvatures of the primary mirror and secondary mirror deviate from the design values due to thermal expansion or contraction. More importantly, due to the non-uniform temperature distribution of the mirror, the displacement caused by temperature changes at each point is also different. Therefore, in addition to uniform expansion or contraction, irregular deformation occurs. The complex deformation brings optical aberrations that are difficult to eliminate, thereby reducing the image quality.

Optical aberration analysis can be completed by fitting the distorted surface using Zernike polynomials [8]. Reference [9] analyzed the aberration and image quality under given temperature conditions. Based on the simulation results, the temperature level and allowable temperature differences that the opto-mechanical structure need to maintain were provided. In practice, the temperature distribution is influenced by the various heat transfer modes, making it difficult to pre-determine [10]. In addition, simulation techniques need to be improved to comprehensively and accurately evaluate aberrations and image quality [11,12]. Therefore, in product design, it is common practice to directly prescribe the working temperature of the opto-mechanical structure, based on the task requirements and the difficulty of thermal control and considering previous experience, including the average temperature of the primary mirror and secondary mirror, the temperature difference between them, and their own temperature uniformity. For aerial opto-mechanical structures, the temperature is also affected by the complex convection, which makes uniformity very difficult. Therefore, the axial/radial temperature differences within the mirrors, as well as the temperature difference between the primary mirror and secondary mirror, are generally limited to 5 °C, while the average temperature fluctuates slightly around 20 °C [2,13].

Due to the significant temperature changes in the environment where the EO system operates, thermal control design must be carried out to ensure that the opto-mechanical structure, especially the primary mirror and secondary mirror, meets the temperature requirements during the working cycle. When the mirror is composed of silicon carbide (SiC), due to its large thermal conductivity, temperature uniformity is easily satisfied. The fused silica mirror, while offering great benefits, such as mature and low-cost manufacturing and hence wide usage, presents also great challenges: it has poor thermal conductivity, which makes temperature uniformity difficult. It is necessary to reduce the heating power to ensure temperature uniformity, but this will reduce the ability to raise the temperature.

To guarantee the temperature uniformity of the fused silica mirror, this article analyzes the relationship between the conventional heating method and axial/radial temperature differences, and an optimized thermal control design based on temperature-negative feedback-variable power zonal heating is proposed. Through this thermal control technology, the axial/radial temperature differences can be controlled within the threshold. At the same time, it maintains a balance between the heating power and heat losses, ensuring that the temperature is at an appropriate level during the working cycle, thereby almost eliminating the changes in curvature radius and aberration caused by temperature changes and guaranteeing the good image quality of the EO system.

## 2. Typical Aerial Opto-Mechanical Structure and Its Heat Transfer

**Figure 1:**
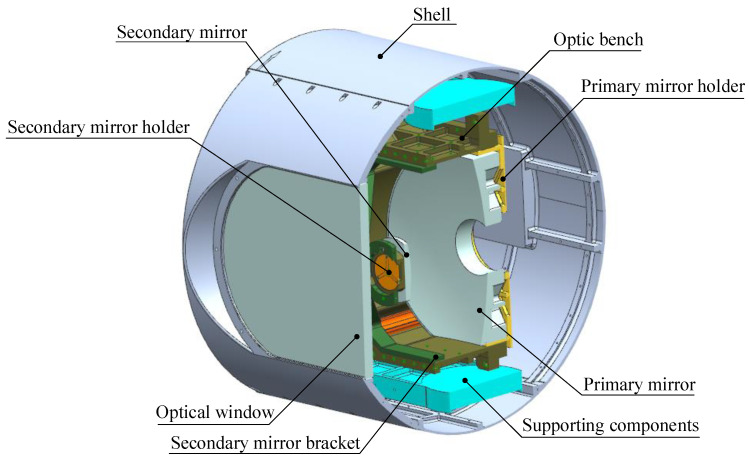
Structure of reflective opto-mechanical structure.

A typical aerial opto-mechanical structure is shown in Figure 1. The primary mirror and secondary mirror are mounted on the primary mirror holder and secondary mirror holder, respectively, and they are connected through the optic bench and secondary mirror bracket to form the main body of the reflective opto-mechanical structure. The opto-mechanical structure is connected to the shell of the EO through supporting components and is surrounded by a cabin formed by the shell and the optical window, which is usually cylindrical or spherical.

As the flight altitude and endurance of the aircraft increase, the opto-mechanical structure will undergo long-term heat exchange with low-temperature environments, resulting in a decrease in its own temperature. Firstly, conduction occurs between the opto-mechanical structure and the supporting components, and the heat is ultimately transmitted to the shell. The primary mirror and secondary mirror are bonded to the primary mirror holder and secondary mirror holder through silicone rubber, which is insulative and has a small bonding area. Therefore, the heat losses of the primary mirror and secondary mirror by conduction are very small. For the optic bench and support components, with contact surface temperatures of *T*_ob_ and *T*_sc_, the heat rate by conduction is calculated according to Formula (1):

(1) $$Q_{\mathrm{cond}}^{\mathrm{ca}}=A_{\mathrm{ca}}\frac{T_{\mathrm{ob}}-T_{\mathrm{sc}}}{R_{\mathrm{cont}}}$$

In Formula (1), *A*_ca_ is the contact area between the two, and *R*_cont_ is the contact thermal resistance. Generally, *A*_ca_ is small while *R*_cont_ is large.

Secondly, the cabin is filled with air and undergoes heat exchange with the opto- mechanical structure through convection, transferring heat to the shell and the optical window and ultimately diffusing it to the external environment. Taking a typical aerial primary mirror as an example, the reflective surface is approximated as a plane, and the normal direction is perpendicular to the direction of gravity. The characteristic size (usually the aperture) is *L* = 300 mm, the temperature of the reflective surface *T*_pm_ = 293 K, and the temperature of the air *T*_a_ = 213 K. After working for a period of time, the temperature of the interior air of the cabin is consistent with the external environment’s temperature (this phenomenon is consistent with reality). The air flow inside the cabin is in free convection, with Raleigh number *Ra_x_*:

(2) $$Ra_{x}=\frac{2g\left(T_{\mathrm{pm}}-T_{a}\right)x^{3}}{\left(T_{\mathrm{pm}}+T_{a}\right)\nu^{2}}Pr=\frac{13.72\left(T_{\mathrm{pm}}-T_{a}\right)x^{3}}{\left(T_{\mathrm{pm}}+T_{a}\right)\nu^{2}},\;x\in \left[0,L\right]$$

In Formula (2), the acceleration of gravity *g* = 9.81 m/s^2^ (at sea level), the Prandtl number of air *Pr* = 0.7, and its kinematic viscosity *ν* is calculated according to the (*T*_pm_ + *T*_a_)/2 condition; it is 11.71 × 10^−6^ m^2^/s. Formula (2) shows that *Ra_x_* ≤ *Ra_L_* = 4.3 × 10^8^ ≤ 10^9^. When the flight altitude becomes higher, *g* decreases (e.g., *g* = 9.74 m/s^2^ at 20 km) and makes *Ra_x_* smaller, meaning that the Raleigh number at any position on the reflective surface and at any flight altitude does not exceed the critical value; therefore, the system displays laminar free convection [14]. Correspondingly, the average convection coefficient $\bar{h}$ of the reflective surface can be calculated according to Formula (3).

(3) $$\bar{h}=\frac{1}{L}\int_{0}^{L}h_{x}dx=\frac{1}{L}\int_{0}^{L}{\left[\frac{g\left(T_{m}-T_{a}\right)x^{3}}{2\left(T_{m}+T_{a}\right)\nu^{2}}\right]}^{\frac{1}{4}}\cdot \frac{k}{x}\cdot f\left(Pr\right)dx$$

where *k* is the thermal conductivity of the air at a temperature of (*T*_pm_ + *T*_a_)/2, which is 22.54 × 10^−3^ W/(m·K); *f* (*Pr*) is a function of the Prandtl number:

(4) $$f\left(Pr\right)=\frac{0.75Pr^{1/2}}{{\left(0.609+1.221Pr^{1/2}+1.238Pr\right)}^{1/4}}$$

By combining Formulas (3) and (4), the $\bar{h}$ of the reflective surface can be calculated to be approximately 5.56 to 5.55 W/(m^2^·K), at sea level and 20 km, respectively. It should be pointed out that there are other criteria involved in deciding whether the heat transfer is laminar or turbulence. There are also different calculation methods for $\bar{h}$, but the results are not significantly different.

The third heat transfer mode of the opto-mechanical structure is radiation. Assuming that all surfaces are diffuse gray surfaces in the analysis, their emissivity *ε* is equal to the absorptivity *α*. *ε* is directionally independent and can be regarded as a constant within the operating temperature range of the EO. Regarding the opto-mechanical structure, it is located within the envelope formed by the shell and the optical window, and its emissivity is *ε*_os_. The absolute temperatures of the opto-mechanical structure and envelope are *T*_os_ and *T*_en_, respectively. Denoting the radiation area of the opto-mechanical structure by *A*_os_, the radiation power $Q_{\mathrm{rad}}^{\mathrm{os}}$ from the opto-mechanical structure to the envelope can be estimated as [15]

(5) $$Q_{\mathrm{rad}}^{\mathrm{os}}=A_{\mathrm{os}}\varepsilon_{\mathrm{os}}\sigma \left(T_{\mathrm{os}}^{4}-T_{\mathrm{en}}^{4}\right)$$

In Formula (5), *σ* = 5.67 × 10^−8^ W (m^2^·K^4^) is the Stefan–Boltzmann constant. Obviously, the higher the value of *T*_en_, the less heat the opto-mechanical structure loses through radiation. When $T_{\mathrm{os}}<T_{\mathrm{en}}$, $Q_{\mathrm{rad}}^{\mathrm{os}}<0$, it means that the opto-mechanical structure is heated. In order to obtain accurate results, the radiation of components such as the primary mirror, secondary mirror, and optic bench must be calculated more precisely, often using numerical methods. However, Formula (5) can be used to qualitatively analyze the trend of radiation heat transfer.

## 3. Analysis of Temperature Uniformity and Optimization of Thermal Control

**Figure 2:**
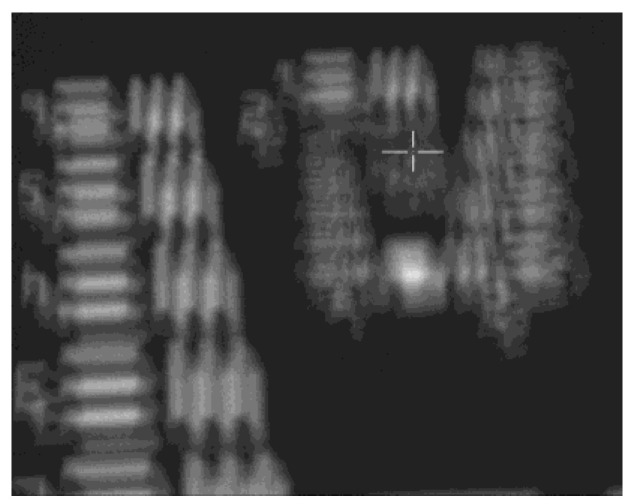
Image quality in low-temperature environment.

The analysis in the second section shows that the heat losses of the opto-mechanical structure occur mainly through convection and radiation. Although electronic units and imaging sensors generate heat during operation, this is not sufficient to compensate for the heat losses of the opto-mechanical structure. Without additional heat input, the temperature of the opto-mechanical structure will ultimately match the ambient temperature. Thus, the image quality may become very poor. Figure 2 shows the resolution test chart of a certain EO system after being stored at −40 °C for 6 h. At this point, the temperature of the opto-mechanical structure is about −35 °C, and clear imaging is impossible. Therefore, it is necessary to heat the opto-mechanical structure, especially the primary mirror and secondary mirror, to ensure the image quality of the EO system.

The conventional heating method for the primary mirror is to arrange a heating zone on its back [16]. The heat starts from the primary mirror holder and reaches the back of the primary mirror through radiation and conduction, and it continues to transmit axially to the reflective surface. This brings about axial/radial temperature differences. The following analyzes the relationship between the temperature uniformity and heating power for reflective faces of different shapes and materials, and optimized thermal control is proposed with the goal of reducing the temperature gradient.

**Figure 3:**
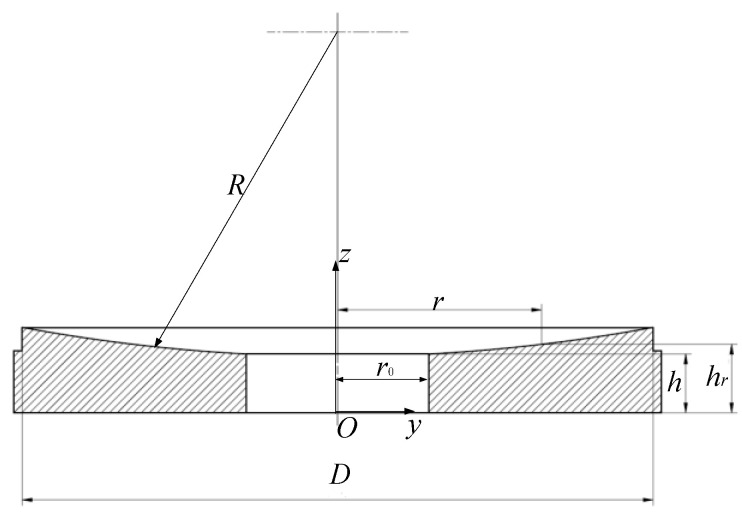
Spherical mirror.

Take a spherical reflective face as an example, as shown in Figure 3. The curvature radius is denoted as *R*, the aperture as *D*, and the center thickness as *h*, and there is a hole with a radius of *r*_0_ in the center of the mirror. The thermal conductivity of the mirror is *k*.

On the reflective surface, at a radial coordinate *r* (0 ≤ r ≤ D/2), the distance *h_r_* from the back is

(6) $$h_{r}=h+R-\sqrt{R^{2}-r^{2}}$$

When the back of the mirror is heated, the vast majority of the heat is transferred from the back to the reflective surface through conduction. The heat transfer rate *q*_cond_ per unit area are shown in Formula (7):

(7) $$q_{\mathrm{cond}}=-k\frac{dT}{dz}$$

Assuming a uniform temperature distribution *T*_b_ on the back, under steady-state conditions, the temperature *T_r_* of the reflective surface at the radial coordinate *r* can be calculated using Formulas (6) and (7):

(8) $$q_{\mathrm{cond}}=k\frac{T_{b}-T_{r}}{h_{r}}\Rightarrow T_{r}=T_{b}-\frac{q_{\mathrm{cond}}}{k}\left(h+R-\sqrt{R^{2}-r^{2}}\right)$$

Therefore, the maximum axial temperature difference Δ*T*_ax_ appears at the outer edge of the reflective surface:

(9) $$\Delta T_{\mathrm{ax}}=\frac{q_{\mathrm{cond}}}{k}\left(h+R-\sqrt{R^{2}-{\frac{D}{4}}^{2}}\right)$$

The maximum radial temperature difference Δ*T*_rad_ caused by conduction can also be obtained through Formula (8):

(10) $$\Delta T_{\mathrm{rad}}=\frac{q_{\mathrm{cond}}}{k}\left(\sqrt{R^{2}-r_{0}^{2}}-\sqrt{R^{2}-{\frac{D}{4}}^{2}}\right)$$

Therefore, based on the temperature difference threshold, combined with Formulas (9) and (10), the maximum allowable heating power can be estimated. Similarly, when the reflective surface is parabolic, *T_r_*, Δ*T*_ax_, Δ*T*_rad_ are, respectively,

(11) $$T_{r}=T_{b}-\frac{q_{\mathrm{cond}}}{k}\left(h+\frac{1}{2}cr^{2}\right)$$

(12) $$\Delta T_{\mathrm{ax}}=\frac{q_{\mathrm{cond}}}{k}\left(h+\frac{1}{8}cD^{2}\right)$$

(13) $$\Delta T_{\mathrm{rad}}=\frac{1}{2}c\frac{q_{\mathrm{cond}}}{k}\left(\frac{1}{4}D^{2}-r_{0}^{2}\right)$$

In Formulas (11)–(13), *c* represents the curvature at the center of the reflective surface, and the meanings of other symbols are consistent with those of spherical mirrors. Similarly, the temperature distributions of other types of reflective surfaces can be analyzed.

From Formulas (9), (10), (12), and (13), it can be inferred that, under the premise of a consistent geometric size and temperature uniformity requirements, the larger the value of *k*, the greater the allowable heating power. For mirrors composed of SiC material, *k*_SiC_ exceeds 100 W/(m·K), so a large amount of power can be applied to the back for heating, allowing the mirror to have a sufficient capacity to raise the temperature, while the temperature gradient is controlled within the specified range. For fused silica mirrors, *k*_fs_ is only 1/100 of *k*_SiC_, so the back heating power must be well controlled to avoid a large temperature gradient. In addition, it is necessary to consider the convection of the reflective surface in reality, and it is difficult to ensure the uniform distribution of *q*_cond_ on the entire back surface. These factors will increase the temperature gradient on the mirror. Therefore, in reality, the allowable maximum heating power is smaller than the estimated value.

Consider a fused silica primary mirror with a spherical reflective surface, *k*_fs_ = 1.37 W/(m·K) (see Table 3.12 of reference [17]), *R* = 600 mm, *D* = 220 mm, *h* = 20 mm, *r*_0_ = 35 mm. When the maximum allowable axial/radial temperature differences are 5 °C, *q*_cond_ = 227 W/m^2^, the maximum heating power of approximately 7.8 W on the back can be applied. As a comparison, let us calculate the maximum allowable heating power of the SiC mirror. *k*_SiC_ is about 150 W/(m·K) (see Table 3.13 of reference [17]), and the corresponding back heating power can be increased to 849 W. Even if the axial/radial temperature differences are limited to 0.5 °C, the back heating power can still be maintained at 85 W. On the other hand, according to Formula (3), when the mirror and air temperatures are 20 °C and −60 °C, respectively, the rate of heat loss is about 16.4 W. This means that when the EO system operates at low temperatures for a long time, in order to maintain the temperature level while ensuring temperature uniformity, mirrors composed of SiC material need heating on the back only [4,16]. For fused silica mirrors, however, in addition to back heating, more optimized heating measures must also be introduced to maintain the temperature level.

**Figure 4:**
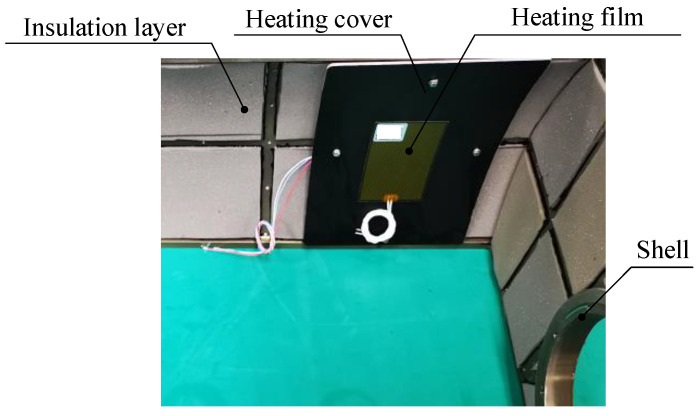
Shell thermal control.

**Figure 5:**
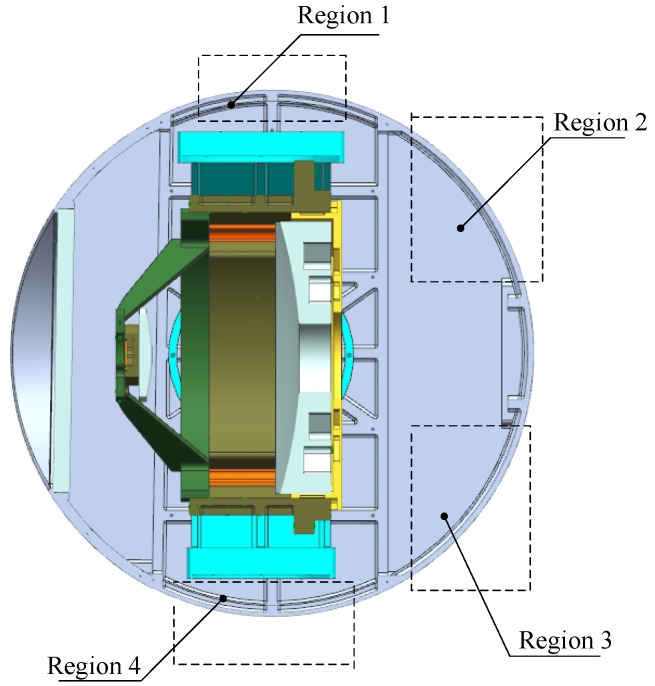
Shell zonal thermal control.

One optimization measure is to arrange thermal control devices on the shell, including passive insulation and active heating, as shown in Figure 4. An insulation layer with low thermal conductivity is bonded to the interior surface of the shell, reducing the rate of heat loss from the inside of the EO to the external environment. The heating cover, a metal sheet with large thermal conductivity, is connected to the shell. The heating cover is attached with a heating film. The heating film is a sandwich structure with resistive heating wires wrapped in polyimide film; it has an operating temperature range of −70 °C to 125 °C, and the temperature coefficient of resistance (change in resistance with temperature) does not exceed 6.18 × 10^−5^ Ω/°C. The heat generated by the heating film is evenly distributed on the heating cover, heating the opto-mechanical structure in a radiative manner. The heating film works with the temperature sensor. The temperature sensor is accurate to ±0.5 °C and has an operating temperature range of −55 °C to 125 °C; it is bonded to the back of the heating cover and measures the temperature. In addition, considering that convection inside the cabin leads to high temperatures in the upper part and low temperatures in the lower part, active heating is divided into multiple zones, with each zone having independent power control, as shown in Figure 5. The heating power of Region 1 and Region 2 is lower than that of Region 3 and Region 4. When the cabin rotates, Region 1 and Region 2 will turn down, and Region 3 and Region 4 will turn up; the heating power of the corresponding area will be increased or decreased accordingly.

**Figure 6:**
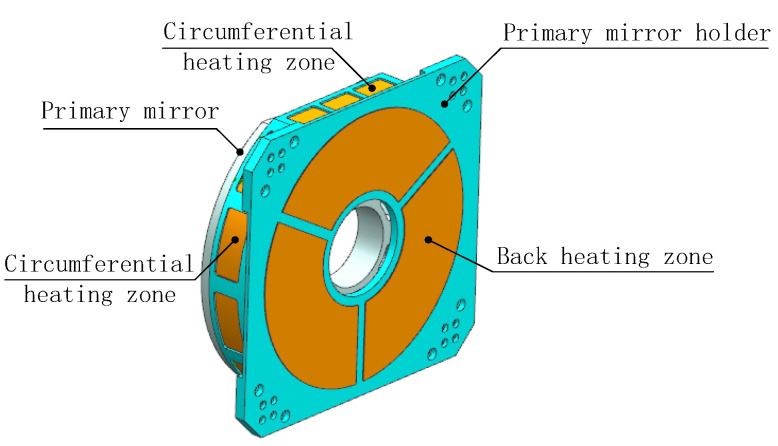
Optimized primary mirror heating.

**Figure 7:**
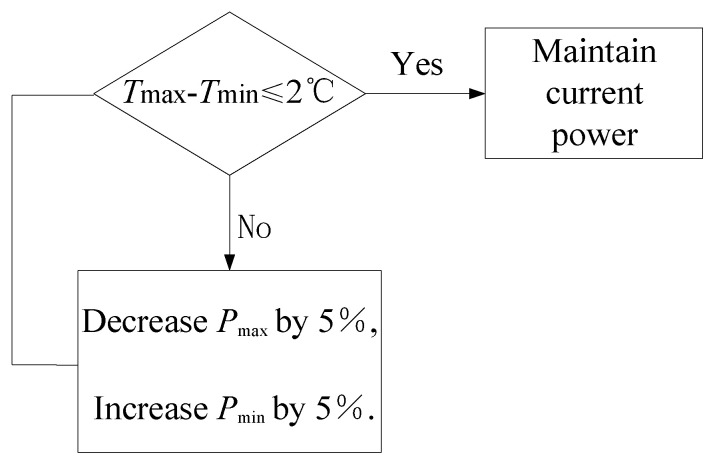
Primary mirror thermal control scheme.

From Figure 5, it can be observed that the view factor of the shell with respect to the primary mirror and secondary mirror is relatively small, indicating that the ratio of the radiation energy leaving the shell that is intercepted by the primary mirror and secondary mirror is small. If a heating zone is set in front of the primary mirror (such as the front part of the shell or the front part of the optic bench [8,16]), although the view factor can be increased, it will cause an uneven air temperature in front of the primary mirror, leading to fluctuations of the index of refraction and reducing the image quality. To improve the heating efficiency and ensure the image quality, heating films are arranged in the circumference of the primary mirror holder, not protruding beyond the front edge of the primary mirror along the axis direction, as shown in Figure 6. As with shell heating, the circumferential heating of the primary mirror holder is also divided into multiple zones, and sensors are arranged on the back and outer edge of the mirror to measure the temperature. Considering the wide range of environmental temperature changes in practical use, the heating power of each zone is independently controlled through temperature-negative feedback to obtain dynamic temperature adjustment and meet the thermal control needs within the working cycle. At a certain moment, the maximum value of the measured temperature is denoted as *T*_max_ and the minimum value as *T*_min_. Correspondingly, the zonal heating powers are *P*_max_ and *P*_min_, the resistance values of the heating films are *R_i_* and *R_j_*, and the input voltages are *U_i_* and *U_j_*, respectively. Considering thermal inertia, when *T*_max_ − *T*_min_ ≥ 2 °C, the heating powers are adjusted by increasing *U_j_* and decreasing *U_i_*. Since *P*_max_ = (*U_i_*)^2^/*R_i_*, *P*_min_ = (*U_j_*)^2^/*R_j_*, as *P*_min_ increases and *P*_max_ decreases, the temperature difference becomes smaller. After 30 s, the temperature is measured, judged, and heating power is adjusted again. The thermal control scheme is shown in Figure 7.

**Figure 8:**
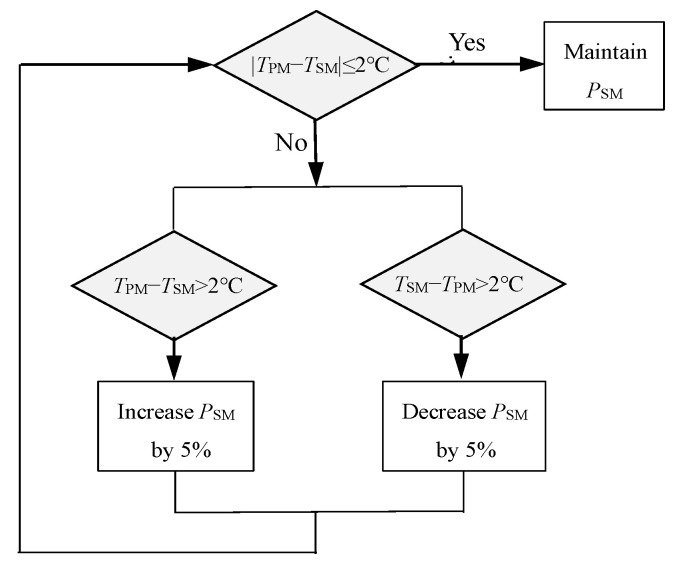
Secondary mirror temperature control scheme.

By conducting the same analysis on the secondary mirror, it is found that due to its smaller size, the convective heat transfer rate $Q_{\mathrm{conv}}^{\mathrm{sm}}$ on the reflective surface is smaller. Under the same temperature uniformity requirements, the allowable heating power *P*_sm_ is larger, which is equivalent to $Q_{\mathrm{conv}}^{\mathrm{sm}}$. Therefore, heating and temperature measurement are set on the back of the secondary mirror only. To control the temperature difference between the primary mirror and the secondary mirror, a thermal control scheme, as shown in Figure 8, is adopted. First, the temperature measured by the primary mirror is averaged as the temperature level *T*_pm_. Secondly, the difference between the secondary mirror temperature *T*_sm_ and *T*_pm_ is determined and *P*_sm_ is adjusted when |*T*_sm_ − *T*_pm_| ≥ 2 °C. The temperature is measured and judged and *P*_sm_ is adjusted again after 30 s.

## 4. Test and Simulation Results

Firstly, the back of the primary mirror is heated and the optimization scheme proposed in Section 3 is used. The temperature rise and uniformity of different thermal control methods are measured and their numerical results are compared. Secondly, thermal control performance tests are conducted on the EO system at a low temperature to measure the temperature and image quality. In Section 4.1 and Section 4.2, *T*_max_ and *T*_min_ are the measured values of the highest and lowest temperatures of the primary mirror, respectively; *T*_max-s_ and *T*_min-s_ are the highest and lowest temperatures of the primary mirror obtained from the simulation, respectively. Finally, the thermal control effect under flight conditions is simulated. All mirrors mentioned in Section 4 are composed of fused silica; the primary mirror holders and secondary mirror holders are composed of titanium alloy; the optical window is composed of K9; and the remaining parts of the opto-mechanical structures are composed of aluminum alloy.

### 4.1. Primary Mirror Heating Test and Simulation

**Figure 9:**
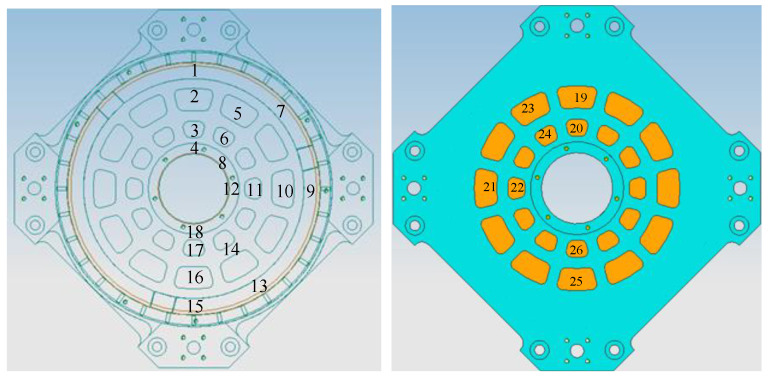
Locations of temperature measurement points.

**Figure 10:**
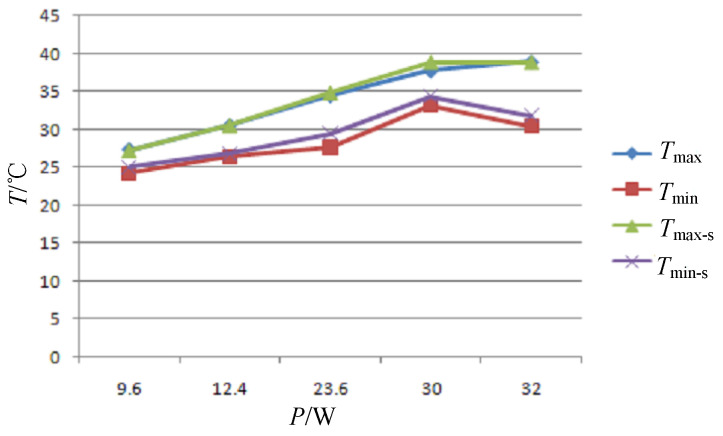
Comparison of measured values and simulation values.

The primary mirror is bonded to the holder and placed vertically; its diameter is 220 mm, its center thickness is 20 mm, and its central hole radius is 35 mm. Eighteen temperature measurement points are arranged on the reflective surface, and 8 temperature measurement points are arranged on the back of the mirror, their numbers and positions are shown in Figure 9. When conducting zonal heating, the total power on the back is 24 W. The circumference is 2 W for the upper part and 4 W for the lower part. The different values of the heating power *P*, axial temperature difference Δ*T*_ax_, radial temperature difference Δ*T*_rad_, and average temperature rise Δ*T* (i.e., the difference between the average temperature of the mirror and the ambient temperature) are shown in Table 1. It should be pointed out that a portion of the heat generated by the heating films undergoes convection with the air, while another undergoes radiation with the environment. The remainder is the effective heating power of the primary mirror. Thus, the values listed in Table 1 are larger than the allowable heating power calculated in Section 3. In addition, due to the vertical position of the mirror, convection will cause the radial temperature difference to be larger than the theoretical value calculated from Formula (10). From the results in Table 1, it can be found that the back heating method allows only a small *p* value to ensure temperature uniformity, which leads to a low capacity to raise the temperature of the mirror. Through optimized zonal heating, larger power can be used to achieve a greater temperature rise while ensuring temperature uniformity. When establishing a thermal analysis model, the heating film is simplified into a two-dimensional sheet, and the heat is evenly distributed on the surface. Features that have a miniscule impact on the temperature, such as small holes and small protrusions, are removed from the model, but the adhesive is retained and the mesh generation is refined. The measured values and numerical results are shown in Figure 10. The numerical results are relatively close to the measured values, with a maximum error of 1.7 °C.

### 4.2. Low-Temperature Testing and Simulation of EO

**Figure 11:**
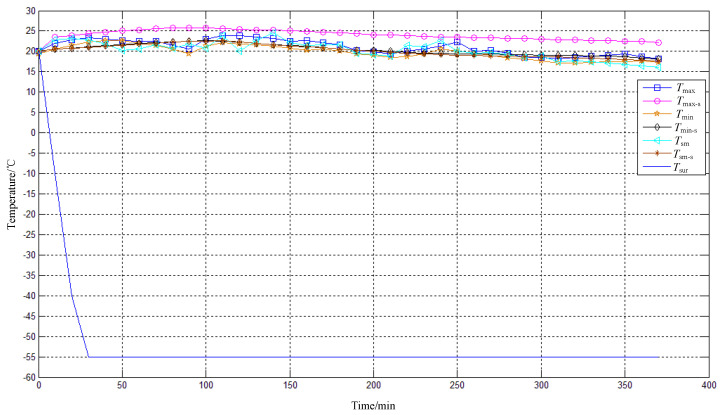
Time–temperature curve (ground test).

**Figure 12:**
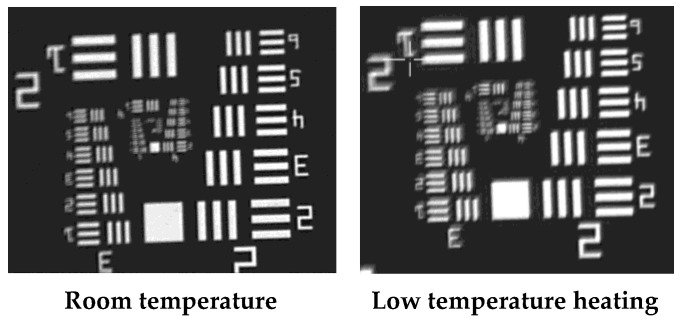
Resolution test.

The optimized thermal control design proposed in Section 3 is applied to the EO system to test the *T*_pm_, *T*_sm_, and image quality at a low temperature (−55 °C). The shell of the EO is cylindrical, and its geometric dimensions are listed in Appendix A (see Table A1). The EO is placed in a chamber with a high–low temperature. The chamber can provide an ambient temperature ranging from −73 °C to 177 °C and has temperature control accuracy of 1.1 °C. The ambient temperature *T*_sur_ drops from 20 °C to −55 °C within 30 min and is maintained for 330 min. The maximum heating power for the upper part of the shell is 110 W, and that for the lower part is 130 W. To ensure convergence, the time step increment of the thermal simulation model is set to 1 s. Due to the fact that the heating of electronic units and imaging sensors has a minimal impact on the temperature of the primary mirror and secondary mirror, they are simplified as uniform heat sources without contact with the opto-mechanical structure. This modeling method can save computational resources and ensure accuracy. Due to the frequent rotation during the experiment, the heating power of the primary mirror and secondary mirror is dynamically adjusted with the attitude of the EO. The time–temperature curve is shown in Figure 11: the temperature of the primary mirror is uniform, and the difference between *T*_max_ and *T*_min_ does not exceed 5 °C. The temperature difference between the primary mirror and the secondary mirror is small. Due to the inability of the model to accurately simulate the rotation and heating power adjustment of the EO, there is a slight error between the numerical results and the test values. Except for *T*_max-s_ − *T*_max_ = 5.2 °C at 90 min, the maximum deviation of the values does not exceed 5 °C, ensuring satisfactory calculation accuracy. The resolution test results at room temperature and a low temperature are shown in Figure 12: the resolution at room temperature is 3.43″, and the system cannot obtain clear images at a low temperature without thermal control (as shown in Figure 2). After using the optimized thermal control, the resolution at a low temperature is 3.82″, which can effectively ensure the image quality.

### 4.3. Thermal Control Simulation under Flight Conditions

**Figure 13:**
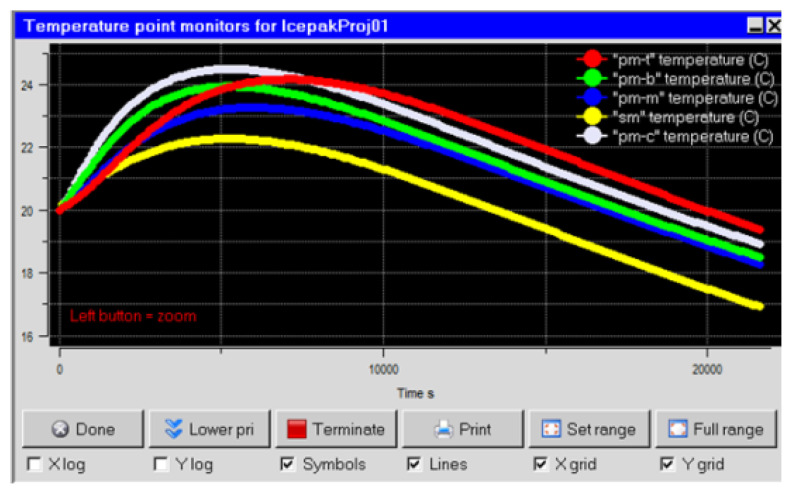
Time–temperature curve (flight conditions).

The EO system mentioned in Section 4.2 is fixed below the belly of the airplane, where the heat exchange with the external low-temperature environment is complex, and the application of the formulas derived in Section 2 and Section 3 will cause significant errors. Therefore, numerical simulation is used to calculate the temperatures of the primary mirror and secondary mirror. The model includes the shell, optical window, primary mirror, secondary mirror, primary mirror holder, secondary mirror holder, optic bench, secondary mirror bracket, supporting component, electronic units, imaging sensors, etc. A thermal simulation model is established using the principles of Section 4.1 and Section 4.2, and densified meshes are adopted to discretize the shell, optical window, primary mirror, and secondary mirror, to ensure the calculation accuracy, totaling 3,672,962 nodes. The initial temperature is 20 °C, and the boundary conditions are set according to the flight speed of 1000 km/h and the flight altitude of 20 km. The heating power is shown in Table 2. The temperature is monitored at the center of the primary mirror, the lower outer edge, the middle outer edge, the upper outer edge, and the center of the secondary mirror, marked as pm-c, pm-b, pm-m, pm-t, and sm in Figure 13. Figure 13 shows that, within 6 h, the average temperature of the primary mirror is not less than 18 °C, its maximum internal temperature difference does not exceed 3 °C, and the temperature difference between the primary mirror and the secondary mirror does not exceed 3 °C. Combining the results of Section 4.1 and Section 4.2, it can be seen that the numerical results have a satisfactory confidence level, and the corresponding thermal control measures can effectively ensure the image quality.

## 5. Conclusions

Through theoretical analysis, temperature and resolution testing, and simulations, the following conclusions are drawn.

(1) Due to the large thermal conductivity, the SiC mirror usually only needs to be heated on the back to ensure that the average temperature is maintained at 20 °C; at the same time, the temperature difference does not exceed the threshold. The thermal conductivity of fused silica is small; thus, it is difficult to balance the temperature uniformity and temperature rise ability of the mirror solely by heating the back only.
(2) By arranging heating zones around the circumference, in addition to back heating, the temperature uniformity of the fused silica mirror can be effectively ensured, while also ensuring that the average temperature is not less than 18 °C. Thus, image quality is ensured, and the resolution of the EO system decreases by no more than 11.4%.
(3) In practical use, according to changes in the environmental temperature and the attitude of the EO, a zonal thermal control design based on temperature-negative feedback-variable power is adopted to fine tune the power of each heating zone, which can effectively improve the low-temperature environmental adaptability of the EO system.

## Figures and Tables

**Table 1:** Power and corresponding temperature.

*P*/W	Back Heating				Optimized Zonal Heating
	9.6	12.4	23.6	32	30
Δ*T*_ax_/°C	1.2	1.4	2.9	3.2	2.5
Δ*T*_rad_/°C	2.8	3.7	6.2	7.5	2.1
Δ*T*/°C	4.3	6.9	9.3	12.9	12.7

**Table 2:** Heating power.

Position	Shell		Primary Mirror		Secondary Mirror
	Upper Part	Lower Part	Back	Circumference	
Power/W	210	240	20	15	5

## Data Availability

All relevant data has been listed in the paper.

## Appendix A

In order to allow the readers to follow up on the design as well as the calculations, the geometric dimensions of the EO and the thermophysical properties of the materials mentioned in Section 4.2 and Section 4.3 are listed in Table A1 and Table A2.

**Table A1:** Geometric dimensions of EO.

Part	Parameter	Magnitude (mm)
Shell	Length	712
	Diameter	524
	Wall thickness	2.5
Primary Mirror	Diameter	312
	Center thickness	29.6
	Central hole radius	40.2
Secondary Mirror	Diameter	94.5
	Center thickness	7.03
	Central hole radius	9

**Table A2:** Thermophysical properties of materials.

Material	Density (kg/m^3^)	Thermal Conductivity (W/m·K)	Specific Heat (J/kg·K)
Fused Silica	2205	1.37	741
Aluminum Alloy	2781	188	921
Titanium Alloy	4452	7.80	678
K9	2510	1.1	879

## References

[1] Yu G., Liu E., Liu G., Zhou L., Zeng J., Chen Y., Zhou X., Zhao R., Zhu S. (2020). Moderate resolution imaging camera(MoRIC) of China’s first Mars mission Tianwen-1. Earth Planet. Phys..

[2] Liu F., Cheng Z., Jia P., Zhang B., Liu X., Hu R. (2019). Impact of thermal control measures on the imaging quality of an aerial optoelectronic sensor. Sensors.

[3] Li S., Wang Y., Zhang H., Yu F. (2020). Thermal analysis and validation of GF-4 remote sensing camera. J. Therm. Sci..

[4] Sun L., Cheng Z., Li L., Liu F., Liu X., Hu R. (2020). Research on precision thermal control technology based on aerial telefocal common aperture photoelectric platform. Opt.-Int. J. Light Electron Opt..

[5] Philip Stahl H., Kuan G., Arnold W.R., Brooks T., Brent Knight J., Martin S. (2020). Habitable-Zone explanet observatory baseline 4-m telescope: Systems-engineering design process and predicted structural thermal optical performance. J. Astron. Telesc. Instrum. Syst..

[6] Lee J.-H., Jung Y.-S., Ryoo S.-Y., Kim Y.-J., Park B.-U., Kim H.-J., Youn S.-K., Park K.-W., Lee H.-B. (2011). Imaging performance analysis of an EO/IR dual band airborne camera. J. Opt. Soc. Korea.

[7] Christine B., Cameryn Y., Janak C., Robert D., Alexander H., Charles H., John L., Alicia M., Samantha W., Shimshone Y. (2023). NASA SAGE SBIR structural, thermal, optical performance(STOP) analysis correlation to wavefront error testing. Proc. SPIE.

[8] Wang D., Li Z., Lin J., Lu M., Li Y., Ran T. (2022). Thermal-optical characteristics analysis of an aerial camera optical system. Appl. Opt..

[9] Liu W., Xu Y., Yao Y., Xu Y., Shen H., Ding Y. (2017). Relationship analysis between transient thermal control mode and image quality for an aerial camera. Appl. Opt..

[10] Holzlöhner R., Kellerer A., Lampater U., Lewis S., Zanoni C. (2022). Structural, thermal, and optical performance analysis applied to subsystems of the European extremely large telescope. J. Astron. Telesc. Instrum. Syst..

[11] Haber A., Draganov J., Krainak M. (2022). Subspace identification of low-dimensional structural-thermal-optical-performance (STOP) models of reflective optics. Proc. SPIE.

[12] Xue Z.-P., Wang C.-X., Yu Y., Wang P.-P., Zhang H.-Y., Sui Y.-Y., Li M., Luo Z.-Y. (2019). Integrated optomechanical analyses and experimental verification for a thermal system of an aerial camera. Appl. Opt..

[13] Li Y., Yuan G., Xie X., Dong L., Yin L. (2012). Multilayer thermal control for high-altitude vertical imaging aerial cameras. Appl. Opt..

[14] Bergman T., Lavine A. (2017). ; Fundamentals of Heat and Mass Transfer.

[15] Howell J., Menguc P., Daun K., Siegel R. (2021). Thermal Radiation Heat Transfer.

[16] Cheng Z., Sun L., Liu F., Liu X., Li L., Li Q., Hu R. (2019). Engineering design of an active-passive combined thermal control technology for an aerial optoelectronic platform. Sensors.

[17] Yoder P., Vukobratovich D. (2015). Opto-Mechanical Systems Design.

